# Intussusception after laparoscopic one anastomosis gastric bypass: A rare complication

**DOI:** 10.1016/j.ijscr.2019.06.014

**Published:** 2019-06-13

**Authors:** Manar A. Al Sulaiti, Abdulla Darwish, Khalid Al Khalifa

**Affiliations:** a Department of General Surgery, Bahrain Defence Force Hospital, Bahrain; b Pathology Department, Bahrain Defense Force Hospital, Bahrain

**Keywords:** Laparoscopic one anastomosis gastric bypass, Intussusception, Bowel obstruction, Case report

## Abstract

**Introduction:**

Intussusception after one anastomosis gastric bypass is a rare postoperative complication that occurs with bowel obstruction. The diagnosis may be challenging because of long-standing, intermittent, nonspecific symptoms. Our paper describes an unusual case of antegrade intussusception that occurred 28 months after laparoscopic one anastomosis gastric bypass surgery.

**Presentation:**

A 30-year-old female known to have diabetes mellitus type 2, who presented with epigastric pain. A computed tomography scan revealed a jejuno-jejunal intussusception. After resection and primary end-to-end hand-sewn anastomosis of the biliary limb, the patient was discharged on the 6th day postoperatively and recovered uneventfully. We belief this is the first report describing a case of intussusception post one anastomosis gastric bypass.

**Discussion:**

Gastric bypass surgeries have recently become a popular method for the surgical treatment of morbid obesity worldwide. The reported intussusception incidence after Roux-en-Y gastric bypass (RYGB) is approximately 0.1–0.3%, but not described in one anastomosis gastric bypass surgery. Approximately two-thirds of patients show recurrent chronic and colicky pain. Surgical intervention is essential.

**Conclusion:**

Clinicians should be aware of such condition in patients with history of bariatric surgery who present with long-standing, intermittent abdominal pain.

## Highlights

• Intussusception is a rare complication of laparoscopic single bowel anastomosis.
• An intestinal polyp is thought to be the leading point of intussusception in this case.
• Post bariatric abdominal pain should not be taken lightly as it can have significant underlying pathology.
• Intussusception in adult regardless of the underlying pathology, surgical intervention is recommended.

## 1 Introduction

Bariatric metabolic surgery has become a favoured procedure for the treatment of morbid obesity and related chronic diseases. Laparoscopic one anastomosis gastric bypass is considered safe and effective, but as with any surgery, there are potential risks and complications, such as diarrhoea, reflex and vomiting; anastomosis complications such as leakage, marginal ulcer and strictures [1].

However, intussusception is rare in adults, accounting for only 5% of all reported cases, and it is rarely seen as a complication after bariatric metabolic surgery [2]. Usually the cause is an underlying pathology in 90%, such as benign or malignant neoplasms, postoperative adhesions, inflammatory bowel disease and congenital abnormalities, such as Meckel’s diverticulum or even iatrogenically [3].

Currently, gastric bypass surgery is widely performed in patients to help in treatment of obesity and related metabolic syndromes. According to Simper, the reported intussusception incidence after Roux-en-Y gastric bypass (RYGB) is approximately 0.1–0.3% [4]. Symptomatic intussusception may also occur many years after surgery [5]. In intussusception, immediate laparoscopy is necessary for all patients who show symptoms, such as peritonitis and signs of perforation or shock. However, in adults, intussusception is rarely observed and is generally caused by pathogenic mucosal, intramural or extrinsic lead points that act as a focal area of traction, pulling the proximal portion of the bowel into the distal portion [6].

Preoperative diagnosis is a challenge in cases of intussusception because of long-standing, intermittent, nonspecific symptoms [7]. Most cases are diagnosed at the time of emergency laparotomy. This paper presents the case of a patient with a past surgical history of laparoscopic one anastomosis gastric bypass who presented with small bowel intussusception of the biliary limb into the enteric limb. While our search for intussusception after gastric bypass surgery revealed many case studies and other types of studies on intussusception following RYGB surgery [8], our paper presents the first case of intussusception after laparoscopic one anastomosis bypass surgery performed in our metabolic and bariatric centre in a tertiary military hospital.

## 2 Case presentation

A 30-year-old Arab female nurse was brought by family to the emergency department with a one-day history of epigastric pain, nausea and vomiting. She did not experience fever or altered bowel habits. Her past medical history revealed a laparoscopic one anastomosis gastric bypass operation for weight loss and diabetes mellitus 28 months prior, within a year her body mass index dropped from 53.6 kg/m [2] to 41.5 kg/m [2] with an estimated weight loss of 40% and was off diabetic medication, as her HbA1c fell from 10.8% to 5.9%. Physical examination showed that the patient was vitally stable but in pain. Abdominal examination revealed a non-distended abdomen with tenderness localized in the epigastric area. Routine laboratory results were unremarkable, and erect and supine abdominal X-rays showed nonspecific signs of intestinal obstruction.

The patient was admitted to internal medicine with an impression of gastritis. She was kept nil per os and given intravenous fluid along with omeprazole 40 mg once per day and metoclopramide 10 mg when necessary, but the condition showed no improvement. The patient was subjective to computed tomography which revealed evident of intraperitoneal free fluid, more significantly multiple dilated proximal jejunal loop. The distal small bowel is rather collapsed denoting small bowel obstruction which is due to jejuno-jejunal intussusception.

**Fig. 1:**
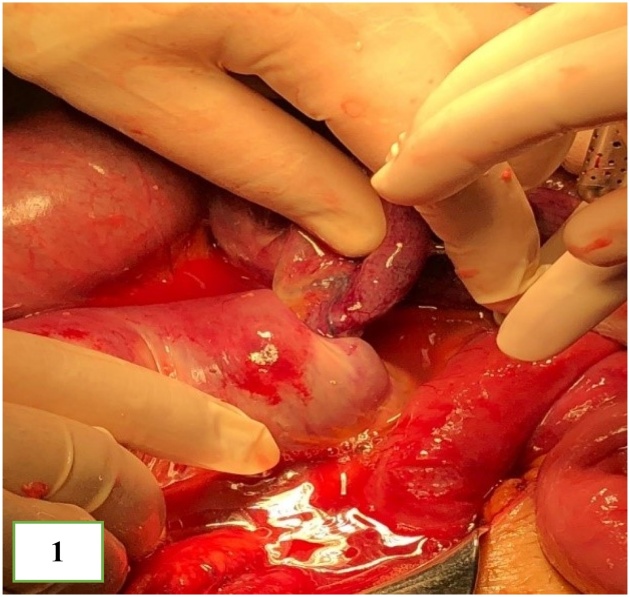
Intraoperative finding of small bowel intussusception of the biliary limb into the enteric limb, with full-thickness wall necrosis.

The general surgical team reviewed the patient and decided to proceed with surgery. The patient initially underwent a laparoscopic approach that was converted to laparotomy due to very large dilation of the small bowel performed by consultant general and a specialized metabolic and bariatric surgeon (Author 3). The intraoperative findings showed a small bowel tight intussusception of the biliary limb into the enteric limb with full-thickness necrosis (Fig. 1). The patient underwent resection, and primary end-to-end hand-sewn anastomosis of the biliary limb just distal to the previous gastrojejunostomy was performed.

The patient was in the intensive care unit for a total of two days observation, kept on antibiotics Piperacillin/Tazobactam 4.5 g and Metronidazole 500 mg, omeprazole 40 mg along with perfalgan 1 g and pethidine 75 mg when necessary all of which are given intravenous except the lateral given intramuscular for a total of 7 days. She was initially kept on Nasogastric tube with nil per so and was gradually proceed to soft diet discharged on the 6th day postoperatively.

Patient was followed up a week after discharge, two weeks then three month and was doing well, with no active complaint.

## 3 Pathology examination

### 3.1 Macroscopic

**Fig. 2:**
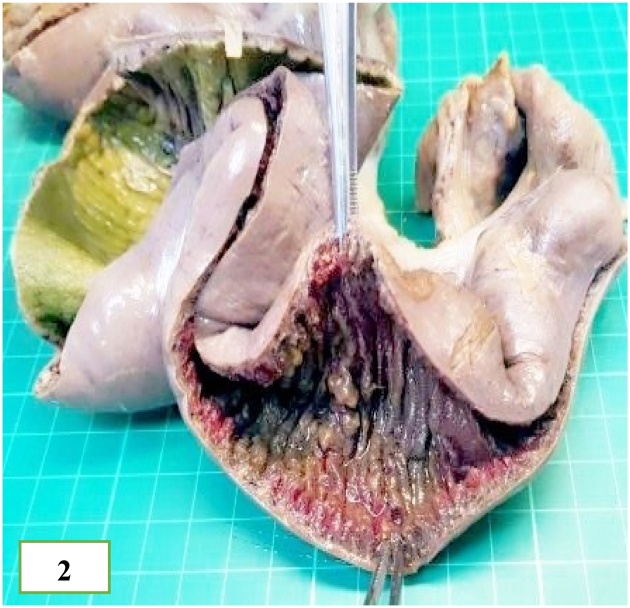
Gross appearance of the resected specimen showing the thickened segment of the small bowel (intussusceptum) on the right side and the dilated part (intususcepiens) on the left side. A hemorrhagic polyp which is partly removed for microscopic examination, is seen at the junction between the thickened and dilated segments. The mucosa is hemorrhagic and the external surface is covered by fibrinoinflammatory exudate indicative of acute peritonitis.

A 55-cm segment of the lustreless, greyish-brown small bowel was examined. The lumen varied from 2.5 to 7 cm in diameter. At 8 cm away from one of the resection margins, an 18-cm narrow segment (intussusceptum) with haemorrhagic, congested mucosa was noted. The dilated segment (intussuscipiens) showed oedematous mucosa with a linear ulcer involving the entire mucosa. A 3 × 1.5-cm haemorrhagic polyp was observed at the junction of the thickened and dilated segments (Fig. 2). The external surface of the specimen was covered by fibrinopurulent exudate, indicative of acute peritonitis.

### 3.2 Microscopic

Surface mucosal ulceration and marked congestion, with haemorrhage of the stroma at the junction of the dilated and thickened segments, were observed. The mucosa adjacent to both the thickened (intussusceptum) and dilated (intussuscipiens) bowel showed mucosal ulceration with mural congestion and haemorrhage, along with dilated lymphatics. The polyp showed haemorrhagic infarction with many congested and dilated vascular channels. The hallmarks of the polyp disappeared before the true polyp type could be identified. The external surface showed features of acute suppurative peritonitis.

## 4 Discussion

Gastric bypass surgeries have recently become a popular method for the surgical treatment of morbid obesity worldwide. After gastric bypass surgery, retrograde intussusception that is not associated with a lead point occurs more often than other types of intussusception. The timely recognition of this condition is increasingly necessary for all medical practitioners because it can prevent considerable morbidities [9]. To date, several theories have been proposed to explain the aetiology of intussusception. The most common theory suggests that the staple/suture line acts as a lead point.

Hyperperistalsis of the excluded segment may telescope the biliopancreatic limb into the common limb. The accumulation of intraluminal fluid in the excluded segment may also force invagination [10]. We believe that the infarcted intestinal polyp found between the two segments represented the leading point in our case. The diagnosis of intussusception in adults is very difficult. The first clinical presentation of intussusception is chronic abdominal pain. This condition usually leads to an incorrect diagnosis [11]. Approximately two-thirds of patients show recurrent chronic and colicky pain. Patients with long-lasting pain in the abdomen should be carefully investigated.

A transient intussusception may be missed on routine examinations. The patient may be dismissed as having a functional problem, which can lead to the delayed treatment of a potential underlying tumour or malignancy. Therefore, associated symptoms, such as nausea and vomiting, need to be considered. According to Azar T, symptoms of intussusception are most often linked to benign underlying tumours, while melaena and guaiac-positive stools are linked to malignant tumours [11].

In conclusion, intussusception is considered a rare complication of gastric bypass procedures. We believe this paper presents the first case of antegrade intussusception following laparoscopic one anastomosis gastric bypass surgery. In conclusion, the clinicians should be aware of such complications, especially when patients present with long-standing, intermittent abdominal pain and a history of bariatric surgery. This work has been reported in line with the SCARE criteria [12].

## Declaration of Competing Interest

The authors declare that they have no conflict of interest.

## Sources of funding

There is no funding for this research.

## Ethical approval

The research and ethic committee at Bahrain Defence Force (BDF) hospital has approved this study.

## Consent

Written informed consent was obtained from the patient for publication of this case report and accompanying images.

## Author’s contribution

Dr. Manar Al. Sulaiti: writing up the paper, she follow up the patient during admission and in clinic.

Dr. Abdulla Darwish: involve in writing and editing the paper and involve in pathological examination of the specimen.

Prof. Khalid Al. Khalifa: the surgeon who performed the procedure and has valuable comments on the manuscript.

## Registration of research studies

researchregistry4940.

## Guarantor

Dr. Abdulla Darwish.

## Provenance and peer review

Not commissioned, externally peer-reviewed.
